# TDP‐43 and APOE: A complex interaction

**DOI:** 10.1002/alz70855_105886

**Published:** 2025-12-24

**Authors:** Muhammad I Abeer, Steven Boone, Michael A. Gitcho

**Affiliations:** ^1^ Delaware State University, Dover, DE, USA

## Abstract

**Background:**

There are currently more than 6 million Americans age 65 or older diagnosed with Alzheimer's disease (AD) which is the seventh leading cause of death in the United States and the most common cause of dementia among older adults [1]. Phenotypically mimicking AD, limbic‐ predominant age‐related TDP‐43 encephalopathy (LATE) accounts for 30‐50% of dementia cases in those older than 80 years of age [2]. Apolipoprotein E epsilon 4 (APOE4) is a major genetic risk factor for AD. Genetic, clinical, and biochemical studies are incomplete in understanding the relationship between APOE4 and TDP‐43 pathology in AD. The overall goal of this study is to investigate the functional relationship between TDP‐43 and APOE.

**Method:**

We utilized primary mouse astrocytes and human hippocampal tissue from AD and LATE cases to study APOE and TDP‐43 interactions. We employed Western blot, co‐immunoprecipitation, immunofluorescence and proteomics to study this interaction. The plasmid for pLX304‐V5 vector (Addgene plasmid #25890) was used as a control and pCMV4‐ APOEe4 was provided from Bradley Hyman (Addgene plasmid # 87087).

**Results:**

We identified an interaction between TDP‐43 and APOE through immunoprecipitation followed by proteomics in mice, verified in human brain by co‐ immunoprecipitation. In addition to that we identified a difference in post translational modifications of APOE between AD patients without TDP‐43 pathology and LATE patients with TDP‐43 pathology. We further show in a cellular model of APOE4 overexpression that endogenous TDP‐43 interacts in complex with APOE4. In addition, primary mouse astrocytes challenged with oxidative stress show a significant increase in endogenous APOE/TDP‐43 colocalization.

**Conclusion:**

Our results indicate a functional relationship between TDP‐43 and APOE which could offer more insights into the pathology of these diseases.

